# Analysis of the Tensile Properties and Probabilistic Characteristics of Large-Tow Carbon Fiber-Reinforced Polymer Composites

**DOI:** 10.3390/polym16152197

**Published:** 2024-08-01

**Authors:** Anni Wang, Ruiheng Li, Xiaogang Liu

**Affiliations:** Research Institute of Urbanization and Urban Safety, School of Civil and Resource Engineering, University of Science and Technology Beijing, Beijing 100083, China; anniwang@ustb.edu.cn (A.W.); liruiheng_rick@163.com (R.L.)

**Keywords:** carbon fiber-reinforced polymer composites rods, carbon fiber-reinforced polymer composites plates, mechanical properties, probability distributions

## Abstract

Large-tow carbon fiber-reinforced polymer composites (CFRP) have great application potential in civil engineering due to their low price, but their basic mechanical properties are still unclear. The tensile properties of large-tow CFRP rods and plates were investigated in this study. First, the tensile properties of unidirectional CFRP rods and plates were studied, and the test results of the relevant mechanical properties were statistically analyzed. The tensile strength of the CFRP rod and plate are 2005.97 MPa and 2069.48 MPa. Second, the surface of the test specimens after failure was observed using a scanning electron microscope to analyze the type of failure and damage evolution process. Finally, the probabilistic characteristics of the mechanical properties were analyzed using normal, lognormal, and Weibull distributions for parameter fitting. Quasi-optimality tests were performed, and a probability distribution model was proposed for the mechanical properties of large-tow CFRP rods and plates.

## 1. Introduction

The density of carbon fiber-reinforced polymer composites (CFRPs) is ~1/5 of the density of steel, and its strength is ~10 times the strength of conventional construction steel. The light weights and high strengths of CFRPs make these materials favorable for civil engineering structures [1]. Depending on the specifications of the carbon fibers, CFRPs can be designed to meet different requirements, such as high strength, high strength and high modulus, and high strength and intermediate modulus [2]. CFRPs have gained prominence in the development of civil engineering structures. In recent years, CFRPs have been widely used for the reinforcement of civil engineering structures, such as carbon fiber cloth reinforcement and prestressed carbon fiber plate reinforcement [3,4,5,6,7]. In addition, owing to the outstanding properties of CFRPs (e.g., light weight, high strength, excellent corrosion resistance, and exceptional fatigue resistance), CFRP cables are being developed to overcome the corrosion–fatigue problem of steel cables, significantly reduce the deadweight of structures, and improve the load-bearing efficiency of large-span structures. For this reason, CFRPs are now being rapidly developed for use in bridges, space structures, and prestressed structures [8,9,10].

Large-scale civil engineering projects have strict requirements in terms of the bearing capacity and stiffness of the structure to meet safety and functional requirements. Therefore, civil engineering materials need to satisfy the dual requirements of strength and elastic modulus. Even though CFRPs have higher strength than conventional steel, their elastic modulus is relatively low, which may affect the deformation and dynamic characteristics of the structure [11]. Increasing the cross-section of CFRPs can improve their stiffness and overcome the problem of an insufficient elastic modulus; however, this affects the ultra-high-strength of the CFRPs, which reduces the cost-effectiveness of the structure. In recent years, large-tow carbon fibers and their composites have garnered much attention from the scientific community. Although the strength of the large-tow carbon fibers is lower than that of conventional carbon fibers, there are no significant differences in the elastic moduli for both of these materials. In addition, improvements in the efficiency of carbon fiber production as a result of the large-tow production process can significantly reduce the cost of carbon fibers and their composites [12,13,14]. Owing to the economic advantages of large-tow carbon fibers, these materials have been widely used in applications such as the main beams of wind turbine blades in recent years [15]. The application of large-tow CFRPs in cables and prestressed tendons can also improve their cost-effectiveness as an engineering material and show broad application prospects in civil engineering structures [16,17].

The tensile properties of CFRPs are the most critical mechanical properties of components such as cables and prestressed tendons. Studies on the tensile properties of conventional CFRPs have confirmed that there is a significant randomness in the tensile strength of these materials [18,19,20]. First, owing to the quality control and process flow during the production process, carbon fibers have a rather random probability of defects. In the process of bearing a continuously increasing tensile load, the random defects in the fibers result in nonnegligible randomness in the tensile strength of the carbon fibers [21]. Second, it is not possible to completely eliminate the random microscopic fiber–resin interface defects or macroscopic defects (such as pores and fiber wrinkles) during the production of CFRPs, which affect their tensile properties [22]. For the above reasons, when CFRPs are subjected to tensile loads, carbon fibers with significant defects will first break due to the high tensile stress, causing a redistribution of shear stress at the fiber–resin interface and a redistribution of tensile stress between the fibers, resulting in a local stress concentration near the broken fibers. When the stress level is sufficiently high, this stress concentration leads to a continuous fracture of fibers, which gradually develops into a fracture of fiber clusters and eventually results in a sudden burst of CFRPs. This tensile failure process is widely known [19,20,23,24,25,26], and existing studies have verified the randomness of the tensile strength of the CFRPs described above. For example, Atadero [27] confirmed that the randomness in tensile strength of CFRPs is influenced by the uncertainty of the fiber or matrix material properties and improper control of the production quality processes.

Owing to the high efficiency of the production process, large-tow carbon fibers offer significant advantages in terms of manufacturing costs and final product prices. However, existing studies have shown that the performance and stability of carbon fibers are also significantly influenced by the production process. In general, small-tow carbon fibers have a relatively higher performance and stability than large-tow carbon fibers [28]. Therefore, the random probability of defects of large-tow carbon fibers may be significantly higher than that of conventional carbon fibers, which affects the tensile properties of large-tow CFRPs. In addition, the fiber surface and sizing qualities significantly affect the fiber–resin interfacial strength [29,30], which affects the clustering development and final burst failure process after the defective fibers break, further affecting the tensile properties of large-tow CFRPs.

Large-tow carbon fiber has great application prospects in civil engineering due to its low price. In designing civil engineering structures, the material strength that satisfies the probabilistic characteristics at a certain confidence level is an important indicator of structural resistance. Therefore, it is essential to study the probabilistic characteristics of material strength [31]. Existing studies have shown that the tensile strength of conventional CFRPs is random, and the characteristics of large-tow carbon fibers may result in more randomness in the tensile strength of large-tow CFRPs. Hence, it is crucial to investigate the probabilistic characteristics of the mechanical properties of large-tow CFRPs. With this in mind, in this study, tensile tests were conducted on large-tow CFRP rods and plates, and the damage evolution and failure processes of these materials were analyzed. In addition, scanning electron microscopy (SEM) was used to examine the cross-section of the CFRP rods and plates post-failure. Following this, the probabilistic characteristics of the tensile properties of large-tow CFRP rods and plates were analyzed, and a probabilistic distribution model was proposed.

## 2. Experimental

### 2.1. Tensile Test of Large-Tow CFRP Rods

T300 carbon fibers were used as the carbon fibers in the CFRP rods, where the tow temperature, fiber volume fraction, diameter, and number of samples were 48 K, 68%, 5 mm, and 32, respectively. The resin used was epoxy. The CFRP rods were formed by pultrusion. Composite pultrusion is a process for producing CFRP profiles by impregnating continuous fibers with resin under the traction of a device and curing the resin by heating a molding. The main steps are as follows:

(1) Carbon fiber yarn arrangement: The carbon fiber on the yarn rack is drawn out from the yarn tube and arranged neatly and evenly.
(2) Impregnation: The neatly arranged carbon fibers are evenly impregnated with the prepared resin.
(3) Molding: The pre-impregnated carbon fiber is initially shaped by a molding device, and after the excess resin is squeezed out, it enters the mold for heating and curing.
(4) Extrusion molding and curing: The impregnated CFRP is made into a rod or plate and heated and cured in the mold.
(5) Traction, winding, and cutting: After the CFRP is pulled out of the mold by the traction device, the profile is collected by the winding device.

**Figure 1:**
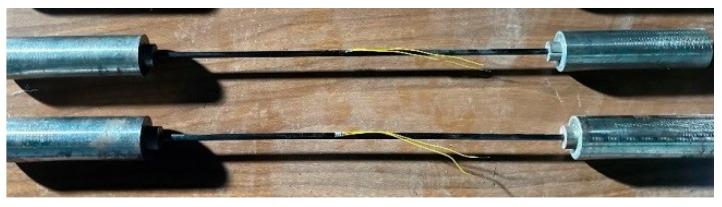
Tensile test specimen of CFRP rod.

The tensile test specimens of the large-tow CFRP rods were prepared according to the specifications of the ASTM D3916 [32] and GB/T 30022-2013 [33] standards. The CFRP rods were anchored with a conical sleeve, with a 0.1° taper difference between the sleeve and clip to relieve the stress concentration at the anchor outlet. The total length of the anchored specimen was ~720 mm, with a free section length of 400 mm and an anchor section length of 160 mm at both ends, as shown in Figure 1. The tensile tests were performed using a 200-kN electronic universal testing machine. The loading speed during the tests was set at 2 mm/min.

The mechanical parameters tested were the tensile strength and longitudinal strain. The tensile modulus was then calculated using Equations (1) and (2), where σ_Gmax_ is the maximum tensile strength (MPa), *F*_Gmax_ is the maximum tensile load (kN), R is the radius of the CFRP rod (mm), *F*_1_ and *F*_2_ represent 50% and 20% of the maximum load (kN), respectively, ε_1_ and ε_2_ represent the strains corresponding to 50% and 20% of the maximum load, respectively, and E_G_ is the tensile modulus (MPa).

(1) $$\sigma_{Gmax}=\frac{F_{Gmax}}{\pi \times R^{2}}$$

(2) $$E_{G}=\frac{F_{1}-F_{2}}{\left(\varepsilon_{1}-\varepsilon_{2}\right)A}$$

### 2.2. Tensile Test of Large-Tow CFRP Plates

**Figure 2:**
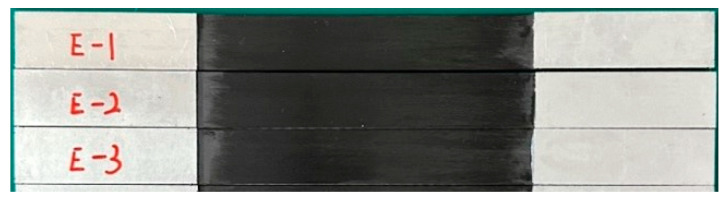
Tensile test specimen of CFRP plate.

The large-tow CFRP plates were pultruded. Tensile test specimens of large-tow CFRP plates were prepared according to the specifications of the ASTM D3039 [34] and GB/T 3354 [35] standards. The length, width, and thickness of the specimens were 310 mm, 25 mm, and 1.5 mm, respectively. Aluminum sheets with dimensions of 80 mm × 25 mm × 2 mm were used as the reinforcement sheets at both ends. After the contact surface between the CFRP plate and reinforcement sheet was lightly polished with sandpaper, 3M DP460 high-strength toughening epoxy resin was used to bond and cure the reinforcement sheet to the CFRP plate, as shown in Figure 2. The loading speed during the tests was set at 2 mm/min. The mechanical parameters tested were the tensile load, transverse strain, and longitudinal strain. Tensile tests were conducted on 30 sets of specimens.

The tensile strength, tensile modulus, and Poisson’s ratio were calculated using Equations (3)–(5). Here, σ_Bmax_ is the maximum tensile strength (MPa), F_Bmax_ is the maximum tensile load (kN), and b and h are the width and thickness of the specimen (mm), respectively. ∆σ and ∆ε denote the tensile stress and strain increments, respectively, and ε_x_ and ε_y_ denote the strain in the fiber direction and the strain perpendicular to the fiber direction, respectively, while μ is the Poisson’s ratio, and E_B_ is the tensile modulus (MPa). It shall be noted that the strain is within a range of 0.001–0.003.

(3) $$\sigma_{Bmax}=\frac{F_{Bmax}}{b\times h}$$

(4) $$E_{B}=\frac{∆\sigma }{∆\varepsilon }$$

(5) $$\mu =-\frac{\varepsilon_{y}}{\varepsilon_{x}}$$

## 3. Results and Discussion

### 3.1. Tensile Test Results of the CFRP Rods

**Figure 3:**
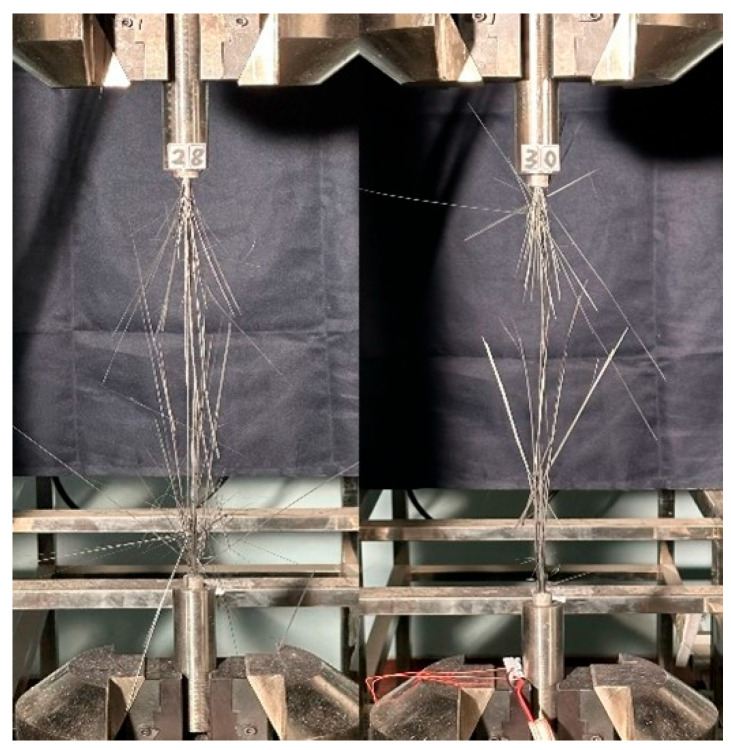
Typical state of CFRP rod after tensile failure.

The tensile test results for the large-tow CFRP rods are summarized in Table 1. Based on the results, the minimum tensile strength was 1605.67 MPa, the maximum tensile strength was 2308.27 MPa, the minimum tensile modulus was 152.73 GPa, and the maximum tensile modulus was 179.26 GPa. Even though the failure modes of the two groups of tests were normal, the effective tensile modulus could not be determined due to the premature failure of the strain gauge. Figure 3 shows the state of a typical CFRP rod after tensile failure. It is apparent that the scattered wires in the middle of the specimen explode, which is a reasonable failure mode.

**Figure 4:**
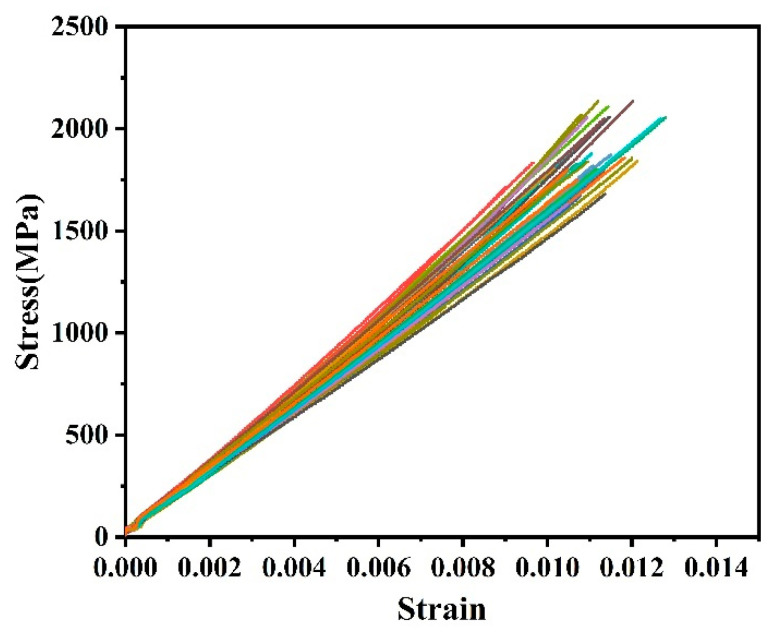
Tensile stress–strain curve of CFRP rod.

Figure 4 shows the tensile stress–strain curves of the large-tow CFRP rods, and it can be seen that there were obvious linear elastic failure characteristics with a certain discreteness. During the tensile tests, when the tensile load exceeded 90% of the ultimate load, the load fluctuated to a certain extent, accompanied by the sound of the fibers breaking. The broken fibers extended from the surface of the rod to the center of the cross-section. When the critical fiber breakage rate was exceeded, explosion failure of the large-tow CFRP rod occurred, accompanied by a loud bang.

### 3.2. Microscopic Analysis of the Tensile Failure of CFRP Rods

**Figure 5:**
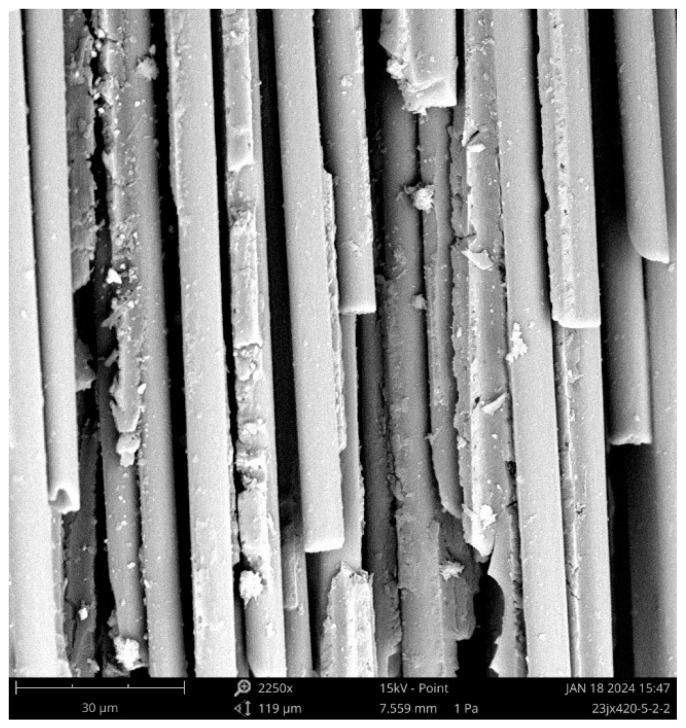
Morphology of CFRP rod after fracture.

SEM was used to analyze the fracture morphology of the CFRP rods. As shown in Figure 5, the residual resin content on the carbon fiber surface was low, and there were large cracks between the fibers and resin matrix attached to the surface. Some of the fibers had smooth surfaces and no residual resin. At the same time, a semicircular resin matrix was observed, indicating that the carbon fibers were pulled out from the resin matrix. Therefore, the interfacial bonding performance between the large-tow carbon fibers and the resin matrix was relatively poor. In addition, the fracture positions of each fiber were uneven, and more flat fiber fractures and some hollow fibers were observed. This indicates that the large-tow carbon fibers may have defects that are densely distributed and are of a large degree, and the performance of the large-tow carbon fibers is relatively low. Based on the fracture morphology, it is perceived that the large-tow CFRP rod first breaks at the larger defect position under the tensile load. Because the fiber–resin interface performance is relatively poor, the internal force redistribution process after the fibers break continuously induces the fiber–resin interface de-bonding failure. Owing to the dense distribution of fiber defects, the adjacent fibers also break at relatively large defect positions. Finally, fine pores were observed on the surface of the local residual resin matrix, and the pore defects affected the tensile properties of the composites.

### 3.3. Tensile Test Results of the CFRP Plates

**Figure 6:**
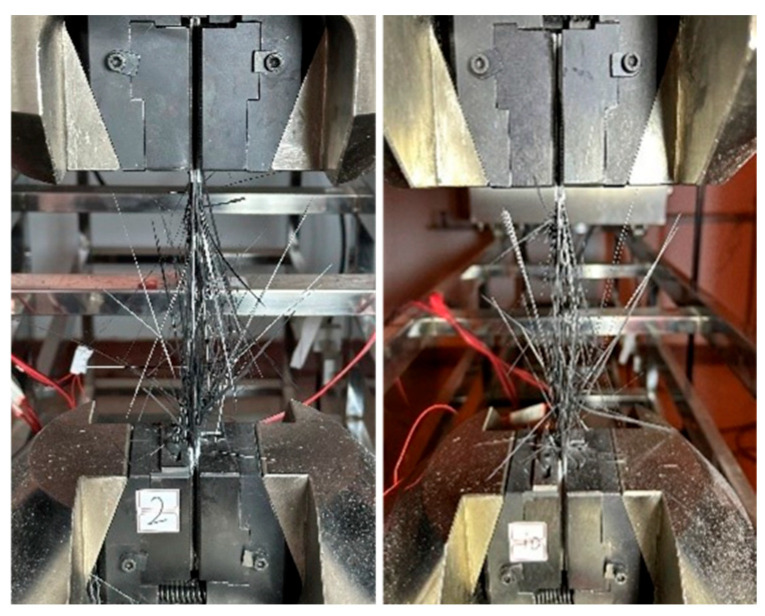
Typical state of CFRP plate after tensile failure.

The tensile test results for the large-tow CFRP plates are summarized in Table 2. The minimum and maximum tensile strength values were calculated to be 1689.91 and 2323.97 MPa, respectively. The minimum and maximum tensile modulus values were determined to be 133.88 and 156.93 GPa, respectively. The maximum and minimum Poisson’s ratios were 0.381 and 0.253, respectively. Although the failure modes of the four groups of tests were normal, the effective elastic modulus and Poisson’s ratio could not be determined due to problems with the strain gauge. Figure 6 shows the state of a typical CFPR plate after tensile failure, and it is apparent that there is explosion damage to the scattered wires in the middle of the specimen, which is a reasonable failure mode. The failure mode of the CFPR plates was more favorable compared with that of the CFRP rods.

**Figure 7:**
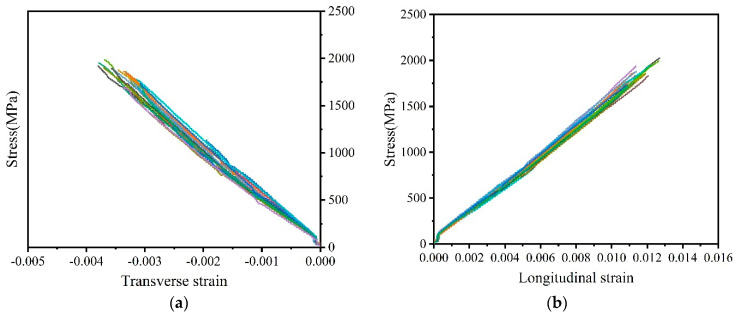
Stress–strain curve of CFRP plate. (**a**) Transverse strain. (**b**) Longitudinal strain.

Figure 7 shows the stress–strain curves of the large-tow CFRP plates. The longitudinal strain in the fiber direction and the transverse strain in the perpendicular fiber direction both revealed obvious linear elastic failure characteristics and exhibited a certain degree of discreteness. However, the degree of discreteness was relatively low for the CFRP plates compared with that for the CFRP rods. During the tests, when the tensile load exceeded 90% of the ultimate load, the tensile load fluctuated to a certain extent. Fiber breakage began at the edge of the CFRP plate and gradually extended to the center of the cross-section. When the critical fiber breakage rate was exceeded, burst failure occurred, accompanied by a loud bang. Before the failure of some of the CFRP plates, the edge fibers did not break at first; rather, burst failure occurred directly. The tensile strength values of the specimens where the burst failure occurred directly were also significantly higher than those of the specimens where the edge fibers broke prior to burst failure. This may be related to the distribution of the microscopic defects in the CFRP plates and the installation accuracy of the test specimens.

### 3.4. Microscopic Analysis of the Tensile Failure of CFRP Plates

**Figure 8:**
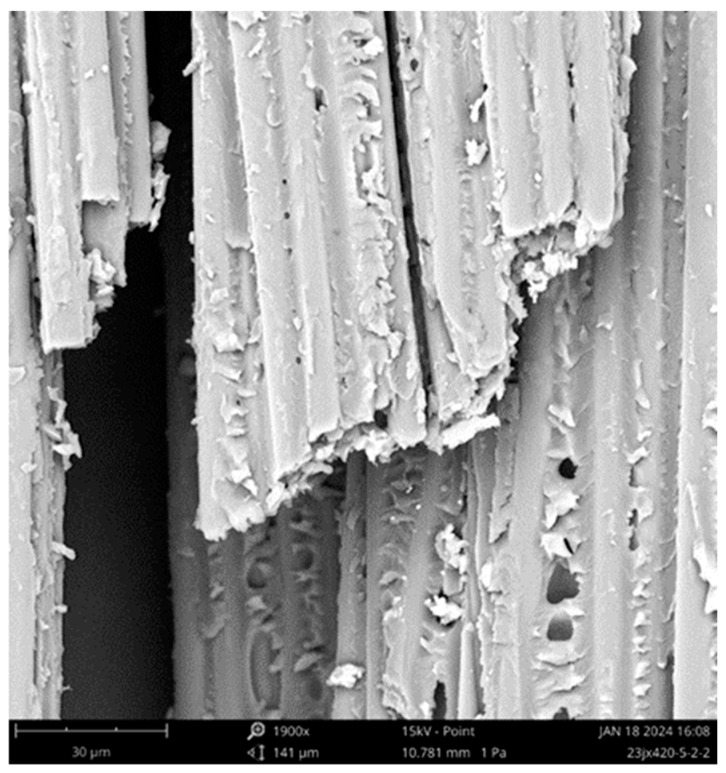
Morphology of CFRP plate after fracture.

Figure 8 shows the SEM image of the tensile failure of a CFRP plate, which indicates a failure mode that is different from that of the CFRP rod. The fiber surface at the fracture of the CFRP plate was still attached to the resin matrix, and there were no smooth fibers completely de-bonded from the resin, indicating that the fiber–resin interface bonding performance of the CFRP plate was better than that of the CFRP rod. In addition, the fracture position of the fiber bundle was more uniform compared with that of the CFRP rod. Based on the fracture morphology of the CFRP plate and its comparative analysis with the CFRP rod, it is perceived that the large-tow CFRP plate first breaks at a larger defect position under the tensile load. However, owing to the relatively good fiber–resin interface performance, the internal force redistribution process after the fibers fracture does not induce continuous fiber–resin interface de-bonding. The fiber–resin interface bonding effectively realizes tensile stress redistribution between the fibers, improves the synergistic force characteristics between the fibers, and also does not cause a dispersed fracture of adjacent fibers at different defect positions. A relatively flat, clustered fiber fracture was observed, which weakened the effect of the fiber defects on the tensile properties of the CFRP plate. This also explains why the tensile strength of the CFRP plate is greater than that of the CFRP rod. Similar to CFRP rods, tiny pores were also observed on the surface of the residual resin matrix at the fracture of the CFRP plate, which influenced the tensile properties of the composite.

### 3.5. Probabilistic Characteristics of the Tensile Properties of Large-Tow CFRP Rods and Plates

The probability distributions of the mechanical properties of materials significantly influence the reliability calculations of components or structures. Different probability distributions may lead to results of different orders of magnitude [36]. Therefore, it is necessary to ensure that the dependence of the probability characteristics on the tail sensitivity is not significant to ensure the robustness of the calculation results [37]. This can be achieved by increasing the number of test samples and performing quasi-optimality tests on the probability distribution. At present, the probability distributions widely used to describe the mechanical properties of composite materials include the Weibull, lognormal, and normal distributions [38]. Therefore, the suitability of these probability distributions was explored in this study, and the Kolmogorov–Smirnov (K–S) and Anderson–Darling (A–D) tests were adopted as the quasi-optimality tests. The fitting results of the tensile properties of the CFRP rods and plates and the K–S and A–D quasi-optimality tests are presented in Table 3 and Table 4, respectively.

#### 3.5.1. Analysis of the Probabilistic Characteristics of Tensile Properties of CFRP Rods

**Figure 9:**
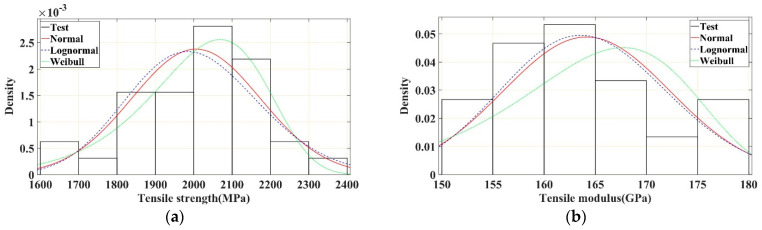
Probability density distribution curve of tensile strength and modulus of CFRP rods. (**a**) Tensile strength. (**b**) Tensile modulus.

**Figure 10:**
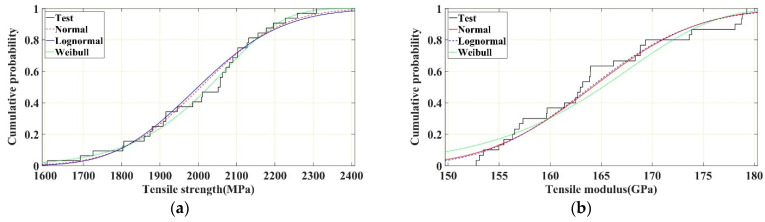
Cumulative probability distribution curves of tensile strength and modulus of CFRP rod. (**a**) Tensile strength. (**b**) Tensile modulus.

The probability density histograms of the tensile strength and tensile modulus of the CFRP rods and the corresponding fitted normal, lognormal, and Weibull distribution curves are shown in Figure 9a,b, respectively. The cumulative probability curves of the tensile strength and tensile modulus of the test specimens and the cumulative probability distribution curves of the fitted normal, lognormal, and Weibull distributions are shown in Figure 10a,b, respectively. It can be observed that the Weibull distribution described the probability characteristics of the tensile strength of the CFRP rods more accurately. S. Gomes et al. [39] and K. Naresh et al. [40] obtained similar conclusions that the Weibull distribution can be used to measure the strength and modulus of CFRP laminates. In contrast, the normal and lognormal distributions described the probability characteristics of the tensile modulus of the CFRP rods more accurately, and the normal and lognormal distributions were nearly coincident with each other. The K–S test and A–D test results of the three probability distributions (Table 3) further verify the results shown in Figure 9 and Figure 10. Based on the K–S and A–D test results, the Weibull distribution is rejected in describing the tensile modulus, indicating that the tensile modulus cannot be represented by the Weibull distribution.

#### 3.5.2. Analysis of the Probabilistic Characteristics of Tensile Properties of CFRP Plates

**Figure 11:**
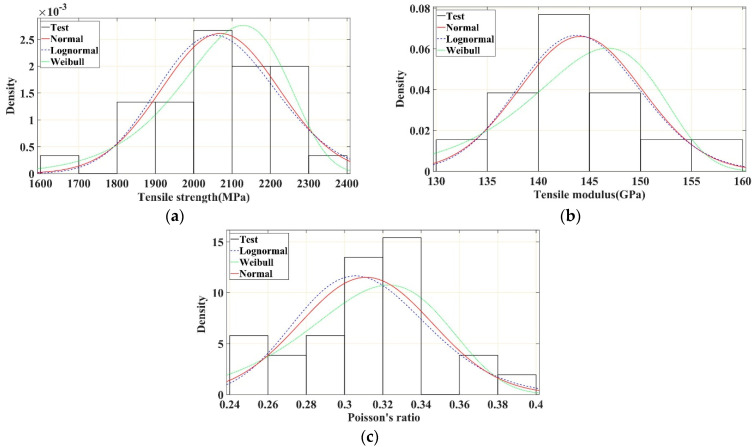
Probability density distribution curves of tensile strength, modulus, and Poisson’s ratio of CFRP unidirectional plate. (**a**) Tensile strength. (**b**) Tensile modulus. (**c**) Poisson’s ratio.

**Figure 12:**
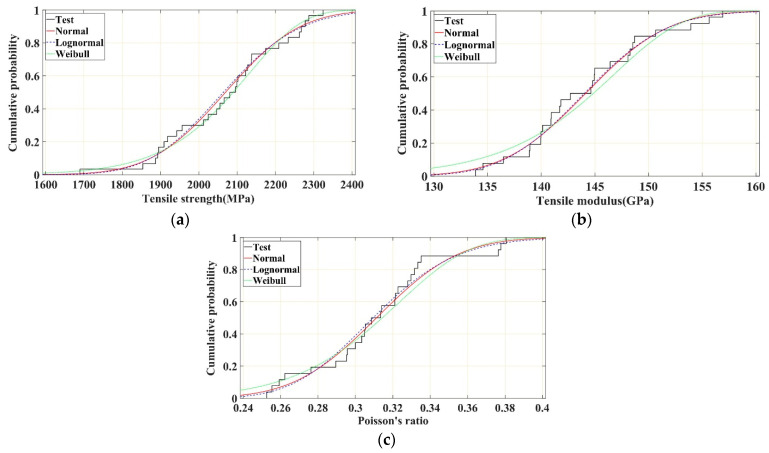
Cumulative probability distribution curves of tensile strength, modulus, and Poisson’s ratio of CFRP unidirectional plate. (**a**) Tensile strength. (**b**) Tensile modulus. (**c**) Poisson’s ratio.

The probability density histograms of the tensile strength, tensile modulus, and Poisson’s ratio of the CFRP plates and the corresponding fitted normal, lognormal, and Weibull distribution curves are shown in Figure 11a–c, respectively. The cumulative probability curves of the tensile strength, tensile modulus, and Poisson’s ratio of the test specimens, and the cumulative probability distribution curves of the fitted normal, lognormal, and Weibull distributions are shown in Figure 12a–c, respectively. It is evident from the results that the normal and lognormal distributions were more accurate in describing the probability characteristics of the tensile strength, tensile modulus, and Poisson’s ratio of the CFRP plates. The lognormal distribution showed a slightly better fit with the aforementioned tensile properties, whereas the Weibull distribution showed an obviously different fit in describing the probability characteristics of the tensile modulus of the CFRP plates. The K–S test and A–D test results of the three probability distributions (Table 4) further verify the results shown in Figure 11 and Figure 12. Based on the K–S and A–D test results, the Weibull distribution is rejected in describing the tensile modulus and Poisson’s ratio of CFRP plates.

## 4. Conclusions

In this study, the tensile properties of large-tow CFRP rods and plates were tested, and SEM was used for the microscopic failure analysis of the test specimens. In addition, the probabilistic characteristics of the tensile properties were analyzed. The following conclusions were drawn based on the key findings of this study:

(1) The tensile failure mode of large-tow CFRP rods and plates shows explosive damage of scattered fibers in the middle of the specimen, which is a reasonable failure mode. The stress–strain curves before failure show obvious linear elastic characteristics. The tensile properties show a certain degree of discreteness, and the discreteness of the CFRP rods is greater than that of the CFRP plates.
(2) SEM images of the surface of the test specimens after failure show that large-tow carbon fibers may have defects that are densely distributed and are of a large degree, and the performance is relatively low. The fiber–resin interface performance of the large-tow CFRP rods is relatively poor, and the fiber–resin interface de-bonds during the tensile process, causing adjacent fibers to continue to break at the location of larger defects, resulting in an uneven fiber fracture. Hence, the tensile strength of the CFRP rods is relatively low. In contrast, the fiber–resin interface performance of large-tow CFRP plates is relatively good, and the bonding effect of the fiber–resin interface effectively improves the synergistic force characteristics between the fibers, presenting a relatively flat, clustered fiber fracture. Thus, the tensile strength of the CFRP plates is higher than that of the CFRP rods.
(3) The Weibull distribution is more accurate in describing the probability characteristics of the tensile strength of the CFRP rods, whereas the normal and lognormal distributions are more accurate in describing the probability characteristics of the tensile modulus of the CFRP rods, and both of these probability distributions are nearly coincident to each other. The normal and lognormal distributions are more accurate in describing the probability characteristics of the tensile strength, tensile modulus, and Poisson’s ratio of the CFRP plates, with the lognormal distribution performing slightly better.

This study only completed the static tensile performance characterization of large-tow CFRPs for pultrusion. In the future, it is necessary to further characterize the mechanical properties and durability of large-tow CFRPs.

## Figures and Tables

**Table 1:** Tensile test results of CFRP rod.

Number	Strength (MPa)	Modulus (GPa)	Number	Strength (MPa)	Modulus (GPa)
1	2072.24	169.33	17	2051.69	163.95
2	1915.60	159.71	18	2177.30	178.75
3	2126.92	153.07	19	1803.25	168.32
4	2090.31	/	20	1908.45	159.63
5	2308.27	156.51	21	2008.46	173.86
6	1691.60	178.82	22	1985.28	161.40
7	1874.15	163.93	23	1605.67	153.47
8	2131.36	179.26	24	2057.07	162.69
9	1805.35	157.33	25	2057.17	168.83
10	2010.48	162.49	26	2258.18	166.19
11	1879.34	156.97	27	2081.19	154.97
12	2102.29	163.84	28	1858.87	168.84
13	1914.81	163.18	29	2155.52	156.30
14	2196.04	178.12	30	1725.21	152.73
15	1945.20	173.63	31	2103.39	162.95
16	2226.95	/	32	2063.36	155.46
average	2005.97	164.15			
variance	27,281.22	64.28			
coefficient of variation	0.08	0.05			

Coefficient of variation: the ratio of the standard deviation to the mean.

**Table 2:** Tensile test results of CFRP plate.

Number	Strength (MPa)	Modulus (GPa)	Poisson’s Ratio	Number	Strength (MPa)	Modulus (GPa)	Poisson’s Ratio
1	2262.38	153.94	0.296	16	2101.76	141.71	0.376
2	2267.35	138.89	0.259	17	2065.89	148.52	0.295
3	2122.24	155.64	0.381	18	1886.93	144.62	0.289
4	1853.71	/	/	19	2137.12	140.13	0.253
5	2080.96	/	/	20	1918.46	136.47	0.332
6	2138.05	144.99	0.309	21	1956.31	150.67	0.305
7	1894.94	148.27	0.378	22	1892.44	148.07	0.330
8	2233.18	140.94	0.314	23	2012.07	142.71	0.262
9	2277.70	156.93	0.335	24	2024.60	144.79	0.303
10	2287.01	146.42	0.314	25	1909.25	/	/
11	2323.97	/	/	26	1942.05	144.94	0.300
12	2099.58	139.98	0.323	27	2174.08	138.95	0.321
13	2054.86	148.70	0.333	28	2174.08	138.95	0.321
14	2046.39	140.90	0.321	29	1689.91	134.56	0.255
15	2208.41	141.82	0.328	30	2127.11	133.88	0.276
average	2069.48	144.13					
variance	22,544.84	34.96					
coefficient of variation	0.07	0.04					

Coefficient of variation: the ratio of the standard deviation to the mean.

**Table 3:** Probabilistic distribution parameter estimation and verification of tensile strength and modulus of CFRP rods.

Properties	Distribution Type	Parameter	Estimated Value	K–S Test	A–D Test	Test Result (α = 0.05)
Tensile strength	Normal distribution	μ	2005.97	0.1074	0.3263	No rejection
		σ	167.81			
	Lognormal distribution	μ	7.60	0.1191	0.4585	No rejection
		σ	0.09			
	Weibull distribution	α	2079.48	0.0802	0.1536	No rejection
		β	14.43			
Tensile modulus	Normal distribution	μ	164.15	0.1432	0.5903	No rejection
		σ	8.15			
	Lognormal distribution	μ	5.10	0.1338	0.5232	No rejection
		σ	0.05			
	Weibull distribution	α	168.11	0.1840	1.0591	A–D test rejection
		β	20.60			

**Table 4:** Probabilistic distribution parameter estimation and verification of tensile strength, modulus, and Poisson’s ratio of CFRP plates.

Properties	Distribution Type	Parameter	Estimated Value	K–S Test	A–D Test	Test Result (α = 0.05)
Tensile strength	Normal distribution	μ	2069.48	0.0720	0.2912	No rejection
		σ	152.72			
	Lognormal distribution	μ	7.63	0.0680	0.3407	No rejection
		σ	0.08			
	Weibull distribution	α	2137.82	0.1006	0.3321	No rejection
		β	16.01			
Tensile modulus	Normal distribution	μ	144.13	0.1107	0.3103	No rejection
		σ	6.03			
	Lognormal distribution	μ	4.97	0.1053	0.2713	No rejection
		σ	0.04			
	Weibull distribution	α	147.06	0.1453	0.7464	A–D test rejection
		β	24.14			
Poisson’s ratio	Normal distribution	μ	0.31	0.1306	0.4685	No rejection
		σ	0.03			
	Lognormal distribution	μ	−1.17	0.1218	0.4572	No rejection
		σ	0.11			
	Weibull distribution	α	0.33	0.1654	0.7741	A–D test rejection
		β	9.48			

## Data Availability

Data are contained within the article.
